# Retrospective Analysis of Liver Enzyme Abnormalities in Patients Prescribed Terbinafine and Itraconazole for Onychomycosis

**DOI:** 10.7759/cureus.44914

**Published:** 2023-09-08

**Authors:** Fatma Etgü

**Affiliations:** 1 Dermatology, Ordu University, Ordu, TUR

**Keywords:** tinea unguium, hepatotoxicity, onychomycosis, itraconazole, terbinafine

## Abstract

Introduction

Onychomycosis (OM) is defined as a nail fungal infection. Its prevalence increases with advancing age. Human-to-human transmission makes it a serious public health risk. Although OM is not a life-threatening disease, it has a detrimental effect on patients’ quality of life. Due to the long therapy duration and potential side effects of systemic antifungal medicines, physicians may be reluctant to treat OM orally. In this study, we aimed to evaluate the effect of terbinafine and itraconazole on liver transaminases, the side effects of these treatments, and patients' adherence to systemic treatment of OM.

Methods

This is a retrospective study conducted in our dermatology department (Ordu University, Ordu) between June 2020 and October 2021. Hospital records were analyzed, and patients with the diagnosis of tinea unguium (ICD code B35.1) were investigated. Patients who were prescribed terbinafine or itraconazole were included in the study. Following a clinical diagnosis of OM, the researchers first tried to confirm it through direct microscopic examination with potassium hydroxide (KOH). If the direct microscopic examination was negative but the suspicion about OM continued, confirmation was done through a fungal culture.

Results

This study included 735 patients, of whom 409 (55.6%) were female and 326 (44.4%) were male. The research covered all of the patients who were given one of these two medications. To find patients who could apply to other hospitals, the Turkish National Healthcare System was checked in addition to hospital information. To identify patients who could apply to other healthcare institutions, all hospitals share their data with this national healthcare system. Terbinafine was used by 433 patients (76.4%), 75 patients (13.2%), and 37 patients (6.5%), respectively, for one, two, and three months. A total of 119 patients (70.8%) took itraconazole for a month, 32 patients (19%) took it for two months, and four patients (2.33%) took it for three months. At the end of the first month, the proportion of the patients with elevated aspartate transaminase (AST) levels was 5.2% for terbinafine and 0% for itraconazole. Eighteen (8.4%) patients with terbinafine had elevated alanine aminotransferase (ALT) levels, and four patients (7.5%) who were on itraconazole treatment had high ALT levels. None of the patients reported cutaneous adverse drug reactions, gastrointestinal disturbances, or headaches due to OM treatment. Also, no patients discontinued treatment because of hepatotoxicity.

Conclusion

In this study, none of the patients discontinued the treatment because of hepatotoxicity. According to the results of this study, oral terbinafine and itraconazole can be used with close follow-up. Baseline and regular laboratory monitoring for AST and ALT should be done to monitor liver toxicity with terbinafine and itraconazole. Besides, we did not observe other side effects like cutaneous or cardiac side effects or drug-drug interactions.

## Introduction

Onychomycosis (OM) is defined as the persistent fungal infection of the nail and nail bed brought on by dermatophytes, non-dermatophyte molds, and yeasts [1-6]. OM is the most common nail disorder, and it accounts for more than half of all onychopathies and 30% of fungal skin infections [1,2]. OM prevalence varies between 0.5 and 14 % in different parts of the world [1,2,5]. OM results in discoloration, thickening, and separation from the nail bed. The most frequently impacted nail is the greater toenail. Males are afflicted more frequently than females [1].

OM prevalence increases with advancing age [1,4,6]. Other factors that increase the risk of developing OM include diabetes mellitus, peripheral arterial disease, psoriasis, immunosuppression, tinea pedis, smoking, recurrent trauma, wearing occlusive footwear, participating in sports, peripheral neuropathies, and traumatic nail disorders [2-4,6]. Human-to-human transmission can happen when people come into direct or indirect contact with objects that have been in contact with the scales or keratin of infected people. This poses a serious public health risk [1].

Although OM is not a life-threatening disease, it has a detrimental effect on patients’ quality of life as its cosmetic appearance can affect psychosocial functioning adversely [1,6-8]. Besides, severely dystrophic nails can result in pain and discomfort and also serve as a reservoir for recurrent fungal and bacterial infections [1,6,7]. Although OM is a curable disease, due to the long therapy duration and potential side effects of systemic antifungal medicines, physicians may be reluctant to treat OM orally [1,7]. Both terbinafine and itraconazole have side effects. Terbinafine may cause headaches, gastrointestinal symptoms, hepatotoxicity, rashes, taste disturbances, and visual disturbances, all of which are self-limiting. Itraconazole has similar side effects to terbinafine, including gastrointestinal distress, headaches, and upper respiratory tract infections [9-13]. Hypertriglyceridemia and elevated transaminases can also develop following itraconazole treatment [12].

OM is generally treated with oral medications since they are cheap, easy to access, and effective. Terbinafine and itraconazole are the most commonly preferred oral antifungals throughout the world due to their high cure rates. The dose for terbinafine is 250 mg, and the dose for itraconazole is 200 mg daily for six weeks for fingernails and 12 weeks for toenails. Itraconazole can be used in pulse regimens [12,13].

In a meta-analysis, it was reported that the risk of asymptomatic elevation of transaminase levels in immunocompetent patients receiving oral antifungal agents was 2%, with only half of the cessation of treatment required [11]. Liver function tests should be checked at the beginning of treatment and after one month of therapy [9].

In this study, we aimed to evaluate the effect of terbinafine and itraconazole on liver transaminases, the side effects of these treatments, and patients' adherence to systemic treatment of OM.

## Materials and methods

This is a retrospective study conducted in the Dermatology Clinic of Ordu University Training and Research Hospital, Ordu, Turkey, between June 2020 and October 2021. Ethical approval was taken before the study. Hospital records were analyzed, and patients with the diagnosis of tinea unguium (ICD code B35.1) were investigated. Patients who were prescribed terbinafine or itraconazole were included in the study. Patients with any known liver or renal diseases who were taking any hepatotoxic drug or who were younger than 18 were excluded from the study. Demographic information about age, gender, baseline, and at the end of the first month of therapy, aspartate transaminase (AST) and alanine aminotransferase (ALT) levels, drug choice (terbinafine or itraconazole), and duration of treatment (month) was recorded. The normal range for AST was 0-32 IU/L, and it was 0-33 IU/L for ALT.

In our clinic, patients were informed about the possible side effects of the drugs, and those who accepted the treatment were prescribed the treatment. As this was a retrospective study, patients were not randomized, and no written informed consent was obtained. All patients who received terbinafine or itraconazole treatment for OM were retrospectively evaluated. The terbinafine dose was 250 mg/day, and the itraconazole dose was 200 mg/day.

In our clinic, the procedure of treating OM begins with the clinical diagnosis of OM, which physicians first try to confirm through direct microscopic examination with potassium hydroxide (KOH). If the direct microscopic examination comes back negative but the suspicion about OM continues, confirmation is done through a fungal culture. Physicians check AST and ALT levels at baseline and the end of the first month of treatment. Venous blood is withdrawn from the patients after at least eight hours of fasting. National health data registries were checked to determine whether patients were requesting general practitioners (GPs) or other physicians to continue treatment for OM. The research covered all of the patients who were given one of these two medications. To find patients who could apply to other hospitals, the Turkish National Healthcare System was checked in addition to hospital information. All hospitals share their data with Turkish National Healthcare. So any patients who applied to other healthcare facilities can be found with this system. Patients with elevated liver enzymes at baseline or during treatment are first consulted by internal medicine, and we only continue the treatment if they give permission. Patients with elevated liver enzymes up to twofold were included in the study. Treatment continues until clinical and mycological cures are achieved.

Statistical analysis

Statistical analysis was performed with IBM SPSS Statistics for Windows, Version 26 (Released 2019; IBM Corp., Armonk, New York, United States). The conformity of the variables to the normal distribution was examined using analytical methods (Kolmogorov-Smirnov and Shapiro-Wilk tests). Descriptive analyses were given as mean±standard deviation and median, min-max for continuous data. To compare terbinafine and itraconazole, the age of the patients was compared using the mean (SD), and the duration of treatment was compared using the median (IQR). Descriptive statistics were made by giving the frequency and percentage values of categorical variables obtained from sociodemographic and clinical information. In continuous data (age, duration of drug use, etc.), the t-test was used for independent groups when it showed a normal distribution to compare binary groups (Terbinafine vs. Itraconazole treatment groups). Pearson’s chi-square or Fisher’s exact test was used for categorical data (AST and ALT normal and high groups). The independent variables were drug groups (terbinafine and itraconazole), age, sex, and duration of treatment, and the outcome variables were AST and ALT levels at the end of the first month. The McNemar Chi-square test was used to compare categorical variables before and after treatment. A p-value below 0.05 was considered statistically significant.

## Results

This study included 735 patients, of whom 409 (55.6%) were female and 326 (44.4%) were male. The mean age of the patients was 47.9±15.1 years, with a minimum age of 18 years and a maximum age of 91 years. The mean age was 45.24±14.73 (range 18 to 78) for women and 51.32±14.96 (range 18 to 91) for males. A statistically significant difference was found between genders (p<0.0001), with males having a higher mean age than females. The mean age for the patients who were under itraconazole was lower than that of the patients who used terbinafine (p<.0001). The mean time that the patients were on drug treatment was not different between terbinafine and itraconazole. The majority of the patients who were on itraconazole treatment were female, and more male patients used terbinafine, but the difference was not statistically significant (Table 1). Terbinafine was used by 433 patients (76.4%), 75 patients (13.2%), and 37 patients (6.5%), respectively, for one, two, and three months. A total of 119 patients (70.8%) took itraconazole for a month, 32 patients (19%) took it for two months, and four patients (2.33%) took it for three months.

**Table 1:** Comparison of gender, age, and duration of drug use among terbinafine and itraconazole

Variables	Terbinafine (n=567)	Itraconazole (n=168)	p Value
	Mean±SD | Median-IQR (min-max)		
Gender (F/M), n(%)	305(53.8)/262(46.2)	104(61.9)/64(38.1)	.063**
Age	49.9±14.9	41.4±14.0	< .0001>
Duration of Drug Use (Month)	1-0 (1-8)	1-1(1-6)	.207*
Independent t-test and *Mann-Whitney U test used for comparing continuous data. Pearson's Chi-square analysis used for categorical data and p < .05 considered significant			

No statistically significant difference was found between the terbinafine and itraconazole treatment groups in the distribution of normal and elevated levels in AST and ALT measurements at baseline (p = 0.362 and p = 0.367, respectively). No statistically significant difference was found between the terbinafine and itraconazole treatment groups when normal and elevated AST and ALT parameters were compared one month after treatment (p = 0.128 and p = 1.000, respectively) (Table 2).

**Table 2:** Comparisons between terbinafine and itraconazole treatments in AST and ALT level groups at baseline and after treatment (one month) AST: Aspartate transaminase; ALT: alanine aminotransferase

AST Level	Baseline			p	After (One Month)			p
	Drug Type		Total		Drug Type		Total	
	Terbinafine (n=567)	Itraconazole (n=168)			Terbinafine (n=211)	Itraconazole (n=53)		
Normal	541(95.4%)	163(97.0%)	704(95.8%)	.362	200(94.8%)	53(100%)	253(95.8%)	.128*
High	26(4.6%)	5(3.0%)	31(4.2%)		11(5.2%)	0(0%)	11(4.2%)	
ALT Level	Baseline			p	After (One Month)			p
	Drug Type		Total		Drug Type		Total	
	Terbinafine (n=567)	Itraconazole (n=168)			Terbinafine (n=214)	Itraconazole (n=53)		
Normal	536(94.7%)	156(92.9%)	692(94.3%)	.367	196(91.6%)	49(92.5%)	245(91.8%)	1.000*
High	30(5.3%)	12(7.1%)	42(5.7%)		18(8.4%)	4(7.5%)	22(8.2%)	
Pearson's or *Fisher's Exact Chi-Square test used and p < .05 considered significant.								

Of the 567 patients using terbinafine, 59 (10.4%) completed the treatment with clinical and mycological improvement, and the treatment was terminated. Of the 168 patients receiving itraconazole, 17 (10.1%) completed treatment. The majority of patients who were prescribed OM treatment-76.4% and 13.2% for terbinafine, 70.8% and 19% for itraconazole-were prescribed once and twice, respectively, and these patients were lost to follow-up. Therefore, the results of this study include not only the patients who completed the treatment but also those who dropped out of the treatment process.

Although 735 patients were included in the study, data from 267 patients with first-month enzyme levels, 72 patients with second-month enzyme levels, 23 patients with third-month enzyme levels, and seven patients with fourth-month enzyme levels could be included in the analysis. When the drug groups (terbinafine and itraconazole) were evaluated together, the rate of patients with elevated AST levels at the end of the first month was 11 (1.5%), and the rate of patients with elevated ALT levels was 22 (3%). At the end of the second month, the AST level was elevated in one patient and the ALT level was elevated in seven patients. At the end of the third month, AST levels were elevated in one patient and ALT levels were elevated in two patients, but AST or ALT levels were not elevated in any of the patients whose results could be obtained in the fourth month.

Baseline ALT levels were above the normal range for 5.3% of the patients on terbinafine treatment and 7.1% of the patients on itraconazole treatment. The ratio of patients with above-normal AST levels was 4.6% for terbinafine and 3% for itraconazole. At the end of the first month, the ratio of patients with elevated AST levels was 5.2% for terbinafine and 0% for itraconazole. Eighteen (8.4%) patients with terbinafine had elevated ALT levels, and four patients (7.5%) who were on itraconazole treatment had high ALT levels. Only one patient who continued terbinafine treatment had elevated AST levels, and six patients (11.8%) had elevated ALT levels at the end of the second month. Thirty-six patients were prescribed itraconazole for two months, and we only reached the enzyme levels of 21 patients at the end of the second month. Of these 21 patients, none had elevated AST levels, and only one (4.8%) had elevated ALT levels. Out of 37 patients who used terbinafine for three months, 19 of them had enzyme results at the end of the third month. Of these 19 patients, one had above-normal AST levels, and two had high ALT levels. None of the four patients who had been on itraconazole treatment for three months had elevated AST or ALT levels. There were seven patients who used terbinafine for four months, and none of them had elevated transaminases. The ratio of patients with high AST and ALT levels was high for males at baseline and the first month (p = .003 for baseline AST and p = .000 for baseline ALT; p = .001 for first-month AST and p = .012 for first-month ALT).

No statistically significant difference was found in the distribution of normal and elevated levels of both AST and ALT parameters obtained at baseline and one month later in the terbinafine treatment group (McNemar test p-value = 1.000). A similar situation was also valid in the itraconazole treatment group. The rates of ALT and AST changes before and after treatment were statistically similar, and no difference was found (Table 3).

**Table 3:** Comparisons between baseline and after treatment (one month) with AST and ALT levels in terbinafine and itraconazole treatment groups AST: Aspartate transaminase; ALT: alanine aminotransferase

AST Level		Terbinafine (n=211)			p	Itraconazole (n=53)			p
		After (One Month)		Total		Drug Type		Total	
		Normal	High			Normal	High		
Baseline	Normal	195(97.5%)	6(54.5%)	201(95.3%)	1.000	51(96.2%)	0(0%)	51(96.2%)	1.000
	High	5(2.5%)	5(45.0%)	10(4.7%)		2(3.8%)	0(0%)	2(3.8%)	
ALT Level		Terbinafine (n=213)			p	Itraconazole (n=53)			p
		After (One Month)		Total		Drug Type		Total	
		Normal	High			Normal	High		
Baseline	Normal	187(95.9%)	9(50.0%)	196(92.0%)	1.000	45(91.8%)	3(75.0%)	245(91.8%)	1.000
	High	8(4.1%)	9(50.0%)	17(8.0%)		4(8.2%)	1(25.0%)	22(8.2%)	
McNemar Chi-Square test used and p < .05 considered significant.									

There were 31 patients with elevated baseline ALT levels. Only one of these patients had more elevated enzyme levels at the end of the first month. In nine patients, enzyme levels decreased, but they were still above the normal range. Twelve patients’ enzyme status was within the normal range. Of the 31 patients with elevated baseline AST levels, two patients had more elevation, three patients had decreased enzyme levels, but they were still above normal, and seven patients had normal enzyme levels at the end of the first month of therapy.

Patients with elevated AST levels at the end of the first month were analyzed. Five of them had normal enzyme levels, and six of them had elevated enzyme levels at baseline. Only one patient had a second-month enzyme level, and it was within the normal range. There were 23 patients with elevated ALT levels at first-month control. Eleven of them had normal enzyme levels at the beginning of treatment. Four had an above-normal enzyme level, which increased more at the end of the first month. In eight patients, enzyme levels were high at baseline; although still high above normal, they decreased.

None of the patients reported cutaneous adverse drug reactions, gastrointestinal disturbances, and headaches due to OM treatment. Also, no patients discontinued treatment because of hepatotoxicity.

## Discussion

For individuals with normal baseline enzyme levels, hepatotoxicity or liver damage is defined as an increase in either the blood alanine transaminase level or the serum aspartate transaminase to more than three times the upper limit of normal. An ALT rise of twice the normal level is reported in patients with preexisting liver impairment, suggesting drug-induced liver damage (DILI) [14]. In this current study, none of the patients experienced hepatotoxicity. Some patients had elevated AST or ALT levels, but they were not twice the normal level. Moreover, the majority of the patients with baseline high AST or ALT levels had decreased enzyme levels, mostly to the normal range in the first month's control. The most frequent toxicity-related reason for the post-marketing withdrawal of a medicine is DILI. The risk of DILI is higher in those with nonalcoholic fatty liver disease, hepatitis C, iron excess, cholestasis, and alcohol use [14].

Terbinafine has been known to cause liver damage, but the precise mechanisms are still unclear [14]. The authors looked at 1,198 individuals who had acute liver failure. DILI was responsible for 133 instances (11.1%), of which six were brought on by antifungal medications, including three linked to terbinafine. The majority of the patients with DILI were female [15]. In a meta-analysis of 122 clinical studies involving 19,298 patients that looked at the safety of oral OM medications, adverse effects caused 3.4%, 2.6%, and 4.2% of patients receiving terbinafine, pulsed itraconazole, and continuous itraconazole, respectively, to stop taking their medications [11]; 1,964 incidences of DILI linked to antifungals were found in a recent pharmacovigilance investigation (FAERS database; 2004-2011), accounting for 2.9% of all DILI cases. One hundred and twelve (5.7%) of these individuals were determined to have liver failure. Terbinafine was blamed for the majority of instances of DILI (n = 422, 27 with LF); the reported odds ratio for liver failure in terbinafine users was 3.39 (95% CI: 2.32-4.96) [16]. According to research, those who had abnormal liver function tests (LFTs) linked to terbinafine were three times more likely to be 65 or older than the rest of the study's participants [17]. Ninety six percent of patients in a comprehensive evaluation of 24 cases of acute liver damage caused by terbinafine were older than 40, and the majority had been using terbinafine for at least three to four weeks [18]. Patients who already suffer from hepatic problems are more likely to have hepatotoxicity [19]. In our study, although some patients had elevated liver enzyme status at the beginning of treatment, no DILI occurred during the follow-up.

In a retrospective analysis of 944 patients receiving terbinafine therapy for OM, AST and ALT elevations alone, respectively, accounted for 92.6% and 91.3% of all liver function test abnormalities. Thus, ALT monitoring and baseline testing by themselves would detect the majority of LFT anomalies and lower related expenditures [17]. Although side effects are not common in patients with OM who were treated with terbinafine, smell and taste disturbances are the most commonly reported side effects [13,20]. None of our patients experienced smell and taste disturbances, cutaneous reactions, or gastrointestinal disturbances in our study.

The FDA has authorized the drug itraconazole for the management of OM brought on by dermatophytes. It can be used daily or in a pulse regimen [21]. In a randomized clinical trial, there was no difference between itraconazole and amphotericin B in terms of hepatotoxicity [22]. Itraconazole had considerably reduced rates of withdrawal due to toxicity and drug-related adverse events in another open, randomized, controlled, multicenter study that compared it to amphotericin B. On the other hand, three patients had to stop taking itraconazole owing to liver dysfunction, whereas no patients receiving amphotericin B discontinued the medication as a result of DILI [23]. In a systematic review of the adverse effects of antifungal agents, the ratio of DILI was 31.6% for itraconazole, 14.1-18.6% for amphotericin, and 1.9% for fluconazole [24]. Authors reported that in patients with itraconazole treatment, the pooled risk of DILI that did not result in discontinuation of the treatment was 17.4% (95% CI: 3.9-31), but the risk of DILI-related treatment withdrawal was 1.5% (95% CI: 0-4) [25]. No patients in our study had to stop their itraconazole treatment because of liver toxicity.

Itraconazole is contraindicated in individuals who have a history of ventricular dysfunction and is linked with congestive heart failure in addition to liver damage. It interacts with a variety of medications by inhibiting CYP34A, which is one of the drug's other significant negative effects. Itraconazole can cause QT prolongation and mortality when used with class IA and class III antiarrhythmics. Rhabdomyolysis might occur if itraconazole is used with HMG-CoA reductase inhibitors. Patients with heart conditions and those at high risk for QT interval prolongation should use itraconazole cautiously. A thorough list of the patients' medications should also be established [9,10,13]. We did not have any patients with cardiac side effects or drug-drug interactions.

Terbinafine inhibits CYP2D6, resulting in a lower risk of drug interaction. Although it has no drug contraindication, physicians should be careful when prescribing terbinafine because the drug metabolizes with CYP2D6 [20]. There were no drug-drug interactions in patients treated with terbinafine or itraconazole.

In a population-based study from Taiwan, oral medications for fungal infections were reported to have a low incidence of acute liver injury; mortal cases were only seen in elderly patients. Also, the risk of liver injury was found to increase with disease duration [26]. In another study, the authors sought to compare the liver toxicity of terbinafine, ketoconazole, itraconazole, fluconazole, and griseofulvin in rats and found that ketoconazole resulted in higher AST and ALT levels. The authors also reported that liver enzymes increased over the long treatment duration of all antifungals, particularly itraconazole and terbinafine [27]. In their analysis, the authors reported more terbinafine-associated liver toxicity in patients who were treated for OM and addressed the fact that a longer duration increases the risk of DILI [28]. It is crucial to regularly monitor liver enzymes in patients who require long-term treatment and in patients who are at risk of developing hepatotoxicity. In our study, we checked the liver enzyme levels of the patients at the beginning of the treatment and regularly throughout the treatment duration. If we found any patients with abnormal liver functions, we consulted internal medicine to ensure a safe treatment procedure.

A potential explanation for the relatively low incidence of hepatotoxicity associated with terbinafine and itraconazole in our study could be attributed to the fact that only 10% of patients successfully completed the prescribed treatment, and the liver enzyme status of patients who were lost to follow-up remained untracked.

Limitations

One of the study's notable limitations pertains to its retrospective nature, which hindered the investigation of potential confounding factors, such as concurrent drug usage or infections, that may have influenced the patients' liver enzyme profiles. Another limitation stemmed from the inability to ascertain the reasons for treatment discontinuation among participants. It is essential to highlight that although the study encompassed a sizable cohort of 735 patients, the availability of enzyme level results at various time points revealed significant discrepancies. Only 36.2% of participants possessed enzyme level data in the first month, with proportions progressively decreasing to 9.8% in the second month, 3.1% in the third month, and a mere 1% in the fourth month. Consequently, caution must be exercised when generalizing the study's findings to the entire participant population.

This investigation primarily aimed to compare the hepatotoxicity of terbinafine and itraconazole. However, a noteworthy limitation emerges from the fact that a substantial proportion of patients did not complete their prescribed treatment, thereby impeding a comprehensive evaluation of the outcomes associated with both drugs.

Despite these limitations, the study boasts strengths, notably its robust sample size. The analysis focused on patients with elevated baseline liver enzyme levels and assessed the impact of treatment on their liver function. Additionally, among patients with elevated liver enzymes at the end of the first month, the study considered their baseline liver status (normal or elevated) and tracked changes in their liver enzyme levels throughout the treatment regimen. Furthermore, efforts were made to evaluate treatment adherence through the national healthcare system.

Recommendation

It is crucial to regularly monitor liver enzymes in patients who require long-term treatment and in patients who are at risk of developing hepatotoxicity.

## Conclusions

OM stands as the most prevalent nail ailment. Despite the apparent safety of terbinafine and itraconazole concerning hepatotoxicity in this study, several critical considerations merit attention. Firstly, merely 10% of patients successfully adhered to the prescribed treatment protocol. Additionally, the proportion of patients subjected to evaluation diminished progressively from 36.2% in the initial month to 9.8% in the second month, 3.1% in the third month, and a mere 1% in the fourth month.

Throughout the study's duration, patients exhibiting abnormal liver enzyme profiles at the outset of treatment or during follow-up received appropriate consultations and close monitoring. Notably, no instances of DILI were observed among patients using either of these medications for a duration exceeding one month. Furthermore, this investigation did not encounter other adverse effects, such as cutaneous or cardiac side effects, or drug-drug interactions.

To monitor liver toxicity when employing terbinafine and itraconazole, it is imperative to conduct baseline and regular laboratory assessments of AST and ALT. OM represents a contagious ailment that significantly impairs patients' quality of life. Therefore, with a thorough medical history and appropriate laboratory testing, oral antifungals can be cautiously employed for the treatment of fungal infections in toenails and fingernails.
